# Dynamics and role of antibodies to *Plasmodium falciparum* merozoite antigens in children living in two settings with differing malaria transmission intensity

**DOI:** 10.1016/j.vaccine.2015.10.058

**Published:** 2016-01-02

**Authors:** David Tiga Kangoye, Victorine Atanase Mensah, Linda Muthoni Murungi, Irene Nkumama, Issa Nebie, Kevin Marsh, Badara Cisse, Philip Bejon, Faith Hope Among’in Osier, Sodiomon Bienvenu Sirima, MVVC Infant Immunology Study Group, Jean-Baptiste Yaro, Siaka Debe, Safiatou Traore, Aminata Ndaw, Babacar Faye, Issiaka Soulama, Amidou Diarra, Alfred Tiono

**Affiliations:** a Centre National de Recherche et de Formation sur le Paludisme (CNRFP), 01 BP 2208, Ouagadougou 01, Burkina Faso; b Service de Parasitologie, Université Cheikh Anta Diop (UCAD), BP 5005 UCAD, Dakar, Senegal; c Kenya Medical Research Institute, Centre for Geographic Medicine Research Coast (KEMRI-CGMRC), P.O. Box 230, Kilifi 80108, Kenya; d Nuffield Department of Medicine, Centre for Clinical Vaccinology and Tropical Medicine, University of Oxford, Churchill Hospital, Oxford, United Kingdom; a Centre National de Recherche et de Formation sur le Paludisme (CNRFP), 01 BP 2208, Ouagadougou 01, Burkina Faso; b Service de Parasitologie, Université Cheikh Anta Diop (UCAD), BP 5005 UCAD, Dakar, Senegal

**Keywords:** Malaria, Infants, Merozoite antigens, Antibody dynamics, Protective threshold

## Abstract

**Background:**

Young infants have reduced susceptibility to febrile malaria compared with older children, but the mechanism for this remains unclear. There are conflicting data on the role of passively acquired antibodies. Here, we examine antibody titres to merozoite surface antigens in the protection of children in their first two years of life in two settings with differing malaria transmission intensity and compare these titres to previously established protective thresholds.

**Methods:**

Two cohorts of children aged four to six weeks were recruited in Banfora, Burkina and Keur Soce, Senegal and followed up for two years. Malaria infections were detected by light microscopic examination of blood smears collected at active and passive case detection visits. The titres of antibodies to the *Plasmodium falciparum* recombinant merozoite proteins (AMA1-3D7, MSP1-19, MSP2-Dd2, and MSP3-3D7) were measured by enzyme-linked immunosorbent assay at 1–6, 9, 12, 15 and 18 months of age and compared with the protective thresholds established in Kenyan children.

**Results:**

Antibody titres were below the protective thresholds throughout the study period and we did not find any association with protection against febrile malaria. Antibodies to AMA1 and MSP1-19 appeared to be markers of exposure in the univariate analysis (and so associated with increasing risk) and adjusting for exposure reduced the strength and significance of this association.

**Conclusion:**

The antibody levels we measured are unlikely to be responsible for the apparent protection against febrile malaria seen in young infants. Further work to identify protective antibody responses might include functional assays and a wider range of antigens.

## Highlights

• Antibody dynamics and role in young the infants’ low susceptibility to febrile malaria were investigated.
• No evidence for association of antibody titres with clinical protection was found.
• Evidence for consistently low antibody titres in high and low transmission areas.
• Other antibodies, other antibody-mediated mechanisms or other protecting factors may be operating.

## 1 Introduction

Evidence of the protective effect of antibodies against *Plasmodium falciparum* febrile malaria was consistently demonstrated in therapeutic passive transfer experiments [1], [2], [3], [4]. Infants passively acquire maternal antibodies, mainly IgG, by placental transfer [5]. Concurrently, transplacental passage of malaria antigens may prime foetal T and B cells and trigger IgM and IgG production [6], although this may be associated with immunosuppression [7]. Infants may be infected [8] but are less likely to develop clinical manifestations of malaria [9] compared with older children. Apart from maternally acquired antibodies, other biological [10], nutritional [11] and physical factors [12], may play an important role in this apparent early and short-lived protection against febrile malaria. Most studies suggest that the reduced susceptibility to malaria lasts until around four months of age [8], [13], [14], [15], [16].

A number of sero-epidemiological field studies have investigated the determinants of naturally acquired immunity in children and adults in various settings with differing malaria transmission levels. These studies have yielded inconsistent associations between anti-malaria antibodies and immunity to malaria [17]. In the investigation of these conflicting results, it has been recently shown that a threshold concentration of antibodies needs to be reached to achieve protection against febrile malaria in children [18], [19].

We recently conducted a study in Burkina Faso examining the impact of maternally-acquired antibodies against synthetic GLURP and MSP3 on the risk of malaria. We found associations between increasing antibody levels and increasing risk of malaria, and no evidence of protective antibody responses. Limitations of this previous study were the limited number of antigens that were examined, and the lack of standardised controls that meant we could not estimate relative antibody concentrations and therefore could not determine how close these antibody levels were to the protective thresholds.

The present malaria sero-epidemiological study expands the range of antibodies examined and includes standardised controls. Furthermore we describe below how antibody levels vary during the first 18 months of life in two settings with differing transmission intensity and we present the results of the analysis testing the hypothesis of an association between total IgG to merozoite antigens and protection against *P. falciparum* febrile malaria.

## 2 Methods

### 2.1 Ethical statement

The ethical approval for the work in Burkina Faso was obtained from the Institutional Review Board of Centre National de Recherche et de Formation sur le Paludisme (CNRFP) in Burkina Faso. In Senegal, the study was approved by the National Ethics Committee. The parents of each child were informed and an individual written consent obtained prior to performing any study-specific procedure on the child. The studies in both settings were conducted according to the principles of the Declaration of Helsinki.

### 2.2 Study site and population

The study was conducted in Banfora, Burkina Faso and in Keur Soce, Senegal.

The Banfora site is described elsewhere [20], [21]. Briefly, the annual rainfall is above 900 mm with the rains lasting from May to October. The transmission of malaria is stable and seasonal. The parasite rates in children aged 2–10 years during the wet and dry seasons in the year preceding the study start were 67.22% and 53.55% respectively. From November 2010 to February 2011,140 children aged four to six weeks were recruited into a prospective longitudinal cohort study. In Keur Soce, the annual rainfall is less than Banfora, at 300 mm with rains from July to October. Transmission of malaria has previously been stable and seasonal, but has recently reduced [22]. The parasite rate in children aged less than ten years and living in Keur Soce was 0.3% in 2010 [23]. A total of 150 infants were recruited into the Keur Soce cohort.

### 2.3 Surveillance of malaria morbidity

In both sites, febrile malaria cases were detected using a combination of active and passive monitoring as detailed elsewhere [21]. The active case detection consisted in scheduled weekly home visits conducted by study nurses to check the children's health status and collect blood films for microscopy when indicated.

### 2.4 Parasitological examination and haemoglobin typing

The identification and quantification of malaria parasites were done as described elsewhere [20]. In brief, each blood smear was examined by two independent microscopists and their respective results were compared for consistency. When their results were concordant, the average was recorded as the final result; otherwise the same blood smear was examined by a third microscopist and the final result was the average of the two most concordant parasite densities. Fractions of foetal haemoglobin and haemoglobin variants were measured using High Pressure Liquid Chromatography.

### 2.5 Selection of samples for serological tests

For a subset of 40 children in each site (sampled randomly without replacement from among all children with complete samples sets available using Stata 13.1), samples at baseline, 2, 3, 4, 5, 6, 9, 12, 15 and 18 months were analysed to compare antibody dynamics. The samples of the remaining children in the Banfora cohort were analysed only at baseline, 3, 6, 9, 12 and 18 months and included in the analysis of the association between antibody levels and risk of clinical malaria in that cohort.

### 2.6 *Plasmodium falciparum* merozoite antigens tested in the assays

The following four recombinant merozoite antigens were tested in this study: the His-tagged AMA1 of the 3D7 allelic form [24], the GST-tagged MSP1-19 of the Wellcome parasite line [25], the GST-tagged MSP2 of the Dd2 parasite line [26] and the MBP-tagged MSP3 of the 3D7 allelic form [27].

### 2.7 Antibody titres measurement by direct ELISA

Serum total IgG titres were measured as described elsewhere [18] with the serum samples diluted at 1:500. The assays testing AMA1-3D7, MSP2-Dd2 and MSP3-3D7 were done in duplicates for the first 372 samples (representing 26% of the total number of samples to analyse) to estimate the variability (coefficient of variation, CV) between the duplicates. We met a pre-set criterion that <5% of sample pairs had a CV > 20 (i.e. 0%, 0.81% and 1.88% respectively for responses to AMA1-3D7, MSP2-Dd2 and MSP3-3D7) and so proceeded with assays in singles rather than duplicates. After adjusting optical densities (ODs) for day to day variation, for antigens that are GST or MBP-tagged, the actual OD of each sample was obtained by subtracting the OD of the tag. A four-parameter logistic regression was used to model the relationship between serial dilutions of a purified IgG standard (Malaria Immune Globulin (MIG) reagent from the Central Laboratory Blood Transfusion Service SRC, Switzerland) of known concentration and the corresponding ODs, and therefore to allow conversion of the study samples ODs into antibody concentrations. Antibody concentrations were then transformed into arbitrary units (AU) as previously described [18].

## 3 Statistical methods

### 3.1 Analysis of the dynamics of antibody titres

We used Pearson correlation test on log-transformed values to measure how strong the relationship is between antibody levels to a given antigen from one time point to the following one and between antigen-specific antibody titres. To estimate the overall decline of antibodies, we used a Random-Effects regression model of antibody titres on age to account for between and within-infant variability of antibody titres. We calculated the cut-off value for seropositivity as the mean OD of negative controls plus 3 standard deviations. Fisher's Exact test was used to compare seroprevalence of antibodies to the *P.f.* merozoite antigens between both the sites at baseline. Fractional polynomial regression models were constructed to fit the non-linear relationship between anti-malaria antibody titre and age. The protective thresholds (for antibodies to AMA1-3D7, MSP1-19, MSP2-Dd2 and MSP3-3D7) displayed in the graphs have been taken from the results of studies conducted by Murungi et al. in the Kenyan Coast [18]. Briefly, they used a modified Poisson regression to model the association between the risk of clinical malaria and antibody concentration categorised into high vs low responders using a series of arbitrary cut-offs within the range of the levels measured in their study children. The protective threshold concentration was then selected based on the log pseudolikelihood. These thresholds have then been validated in a second independent cohort of lower transmission intensity in Kenya [18]. The same methods were used to derive protective thresholds in a children cohort study in Coastal Tanzania [19].

### 3.2 Analysis of correlates of protection

The outcome measure, febrile malaria, was defined as the association of fever (axillary temperature ≥37.5 °C and/or reported fever in the past 24 h) plus asexual parasitemia ≥10,000/μL [21]. An individual malaria exposure index was calculated for each child. Briefly, we have adapted the method of Olotu et al. based on local malaria prevalence and computed instead, individual malaria exposure indexes based on time-to-first malaria infection as described elsewhere [21]. We have investigated the relationship between the putative correlates of protection (seropositivity and antibody titres) and febrile malaria using two approaches. First, we used Cox proportional hazards regression to model the relationship between antibodies and time to first febrile malaria episode, and tested the proportional hazards assumption using the Kaplan Meier method and the Schoenfeld residuals. The second approach consisted of modelling the relationship between antibodies and the number of febrile malaria episodes experienced using a negative binomial regression with the Huber-White Sandwich estimator to account for clustering by individual. In the latter approach, the analysis period was restricted to the three months following each time point for malaria serology to account for the short half-life of anti-malaria antibodies [28]. The antibody titres of the study samples were included in the models as time-changing covariates with the measured value at the beginning of each interval related to the febrile episodes recorded within this interval. The overall significance of categorical variables was estimated using the Wald test. All the antibody titres used in the data analysis are arbitrary units in log scale. The data was analysed using GraphPad Prism version 6.00 for Windows, GraphPad Software and Stata 13.1 for Windows, StataCorp LP.

## 4 Results

### 4.1 Malaria morbidity

The characteristics of the Banfora infant cohort, follow up and malaria morbidity are described in detail elsewhere [21]. Briefly, 296 febrile malaria episodes were recorded over 249 child-years with the number of cases peaking in October each year, giving an incidence rate of 1.2 episodes/child/year (95%CI, 1.06–1.33). In the first six months of life, five infections (two asymptomatic and three febrile) were detected, of which three occurred in the rains, in children aged above five months. In Keur Soce only 4 episodes of asymptomatic malaria were identified in the cohort of 150 infants, and no symptomatic episodes were identified. This is consistent with long-term trends of malaria described elsewhere in Northern Senegal [22].

### 4.2 Variability of antibody titres with time and transmission intensity

**Fig. S1:**
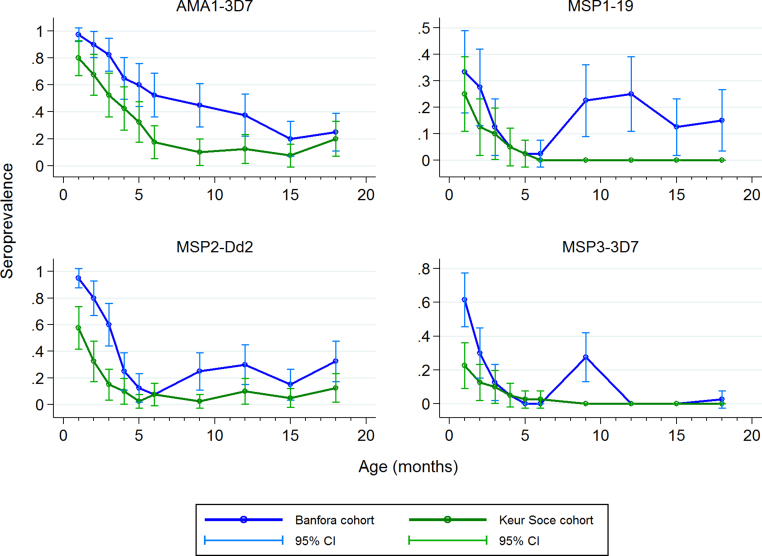
Comparative dynamics of anti-merozoite antibody seroprevalences between Banfora (high transmission intensity) and Keur Soce (low transmission intensity).

**Fig. 1:**
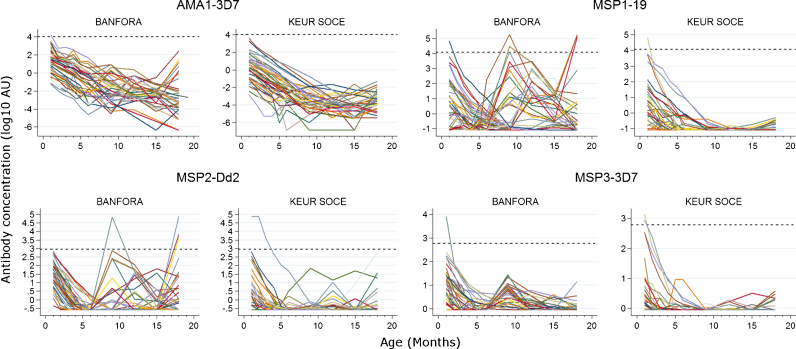
Comparative dynamics of individual antibody titres between Keur Soce and Banfora children. The dashed lines represent the protective thresholds established in children living in the Kenyan Coast. Each line represents an individual trajectory of antibody concentration.

**Fig. 2:**
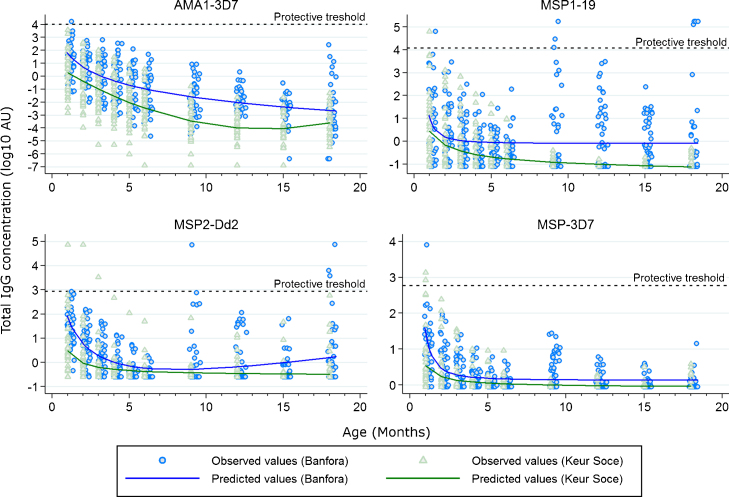
Trend lines of antibodies to *P. falciparum* merozoite antigens. Antibody concentrations are in log_10_ scale.

At baseline, the seroprevalence of the merozoite antigens was significantly higher in the Banfora cohort compared with Keur Soce cohort except for antibodies to MSP1-19 (Table S2). After a steady decline in the first six months of life regardless of the transmission intensity, the seroprevalence to all the antigens tested held below 20% for the remaining monitoring period in Keur Soce, the low transmission area. In Banfora where the transmission is higher, the seroprevalence peaked after six months for MSP1-19 and MSP3-3D7 (Fig. S1). Only anti-MSP2 antibodies increased appreciably from month 9 (Fig. 2). No confidence interval was reported for the protective thresholds; however, the antibody levels in our cohorts were mostly well below the protective thresholds throughout the follow up (Fig. 1). The average antibody decay rates were similar in both the sites only for antibodies to MSP1-19, higher in Banfora for MSP2-Dd2 and MSP3-3D7 and moderately higher in Keur Soce for AMA1-3D7 (Table S3).

Antibody titres to AMA1-3D7, MSP1-19, MSP2-Dd2, MSP3-3D7 were strongly correlated from time point to time point during the first six months of life in both settings regardless of the transmission intensity (*r*, 0.86–0.98 in Keur Soce, 0.86–0.91 in Banfora for AMA-1) (Fig. S3). After the first 6 months of life, antibody titres were weakly to moderately correlated to each other in Keur Soce (*r*, 0.21–0.47) but not correlated in Banfora (−0.21 to 0.03).

### 4.3 Relationship between antibodies and incidence of febrile malaria

#### 4.3.1 Univariate analysis

This analysis applies to Banfora only. In the time-to-event univariate analysis none of the antigen-specific antibodies, in terms of levels or seropositivity, was associated with protection. Antibodies to AMA1-3D7 (HR: 1.34, 95%CI: 1.11–1.62, *p* = 0.002) and MSP1-19 (HR: 1.44, 95%CI: 1.19–1.74, *p* < 0.001) were significantly associated with a higher risk of febrile malaria but season was the strongest significant risk factor for febrile malaria (HR: 8.28, 95%CI: 2.18–31.44, *p* = 0.002). The results of the event count analysis were similar except for anti-AMA1-3D7 antibodies that appeared as significantly associated with protection against febrile malaria episodes (HR: 0.89, 95%CI: 0.80–0.98, *p* = 0.015). Children with haemoglobin CC type had lower risk of febrile malaria compared with haemoglobin AA children. Age was a significantly associated with risk of febrile malaria (Table S4). In both models, the highest level of education was significantly associated with protection and children born in the last quarter of the year had significantly lower risk of febrile malaria compared with those born in January as previously described [21].

### 4.4 Multivariable analysis

**Fig. S2:**
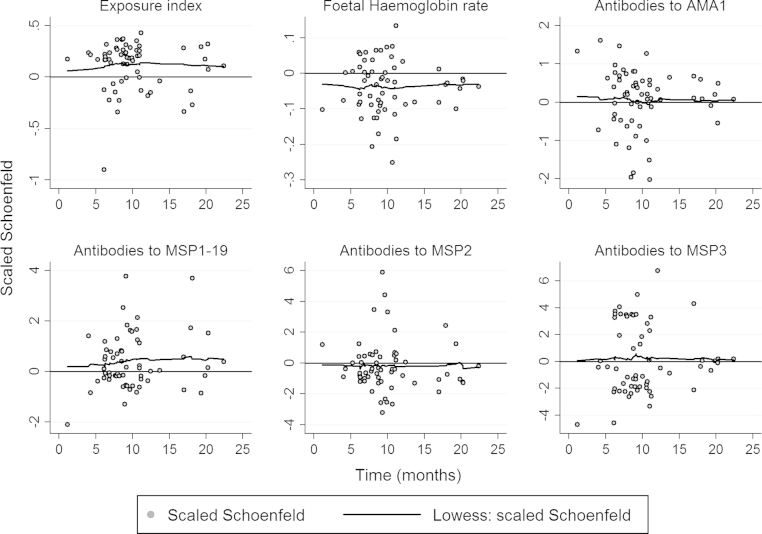
Proportional Hazards assumption test.

**Fig. 3:**
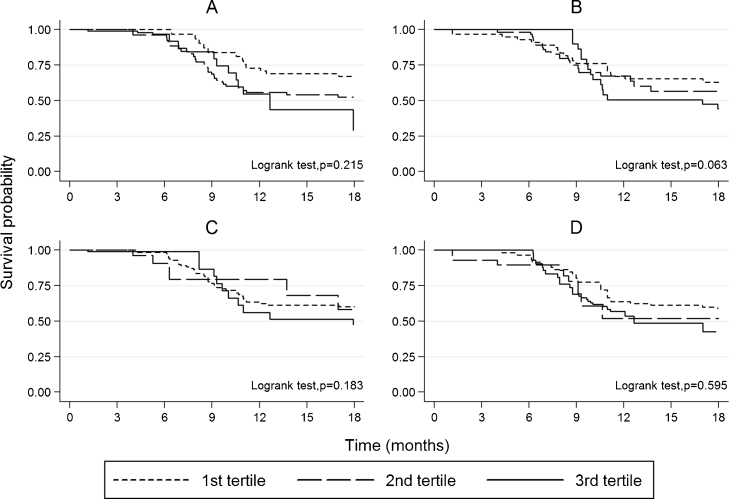
Estimation of children survival in relation to anti-malaria antibody tertiles. (A) antibodies to AMA1-3D7, (B) antibodies to MSP1-19, (C) antibodies to MSP2-Dd2, (D) antibodies to MSP3-3D7.

There was only limited collinearity between the different antibody titres (VIFs < 2; mean VIF = 1.44). In the multivariable time-to-event analysis, foetal haemoglobin rate was weakly but significantly associated with protection (HR: 0.97, 95%CI: 0.94–0.99, *p* = 0.004). Season remained the strongest risk factor (HR: 9.39, 95%CI: 2.32–37.99, *p* = 0.002), and exposure index was associated with a risk of malaria (HR: 1.10, 95%CI: 1.05–1.15, *p* < 0.001). There was a tendency of anti-MSP1-19 antibodies to be associated with higher risk of febrile malaria (HR: 1.40, 95%CI: 1.09–1.80, *p* = 0.008), but overall the correlation between antibodies and increased risk of malaria was reduced by adjusting for exposure and season. There was no significant deviation from the proportional hazards assumption (Figs. 3 and S2). In the event count analysis, none of the antigen-specific antibodies was significantly associated with febrile malaria. Season was confirmed as the strongest risk factor (Table 1). An interaction of weak effect size between season and individual exposure index was observed (IRR = 1.06, 95%CI: 1.002–1.13, *p* = 0.042) in the event count analysis.

## 5 Discussion

The titres of antibodies to all the four antigens tested were below the protective thresholds except for a few outlying results. After a consistent decline up to six months of age, only antibodies to MSP2 showed a steady increase up to month 18 in the high transmission setting (Banfora). Overall, we did not find any protective effect in the investigation of the association between antibodies to *P. falciparum* merozoite antigens and febrile malaria. Antibody titres to some merozoite antigens (AMA1-3D7 and MSP1-19) were rather a maker of exposure to malaria, evidenced by the association with risk and the fact that this association was diminished after adjusting for the exposure index.

**Fig. S3:**
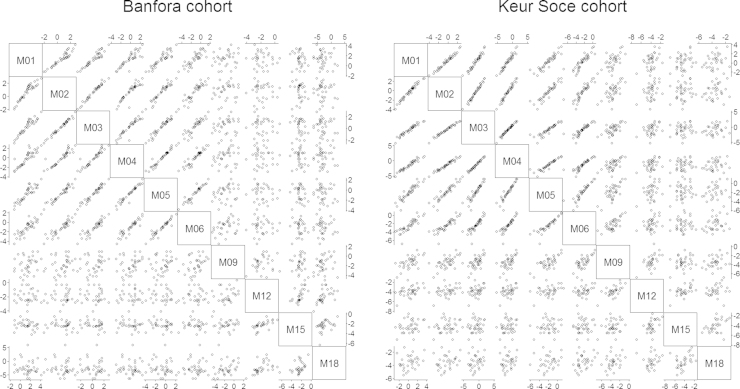
Correlation matrix of antibodies to AMA1-3D7 between time points for antibody titres measurement from baseline to month 18.

Average antibody decay rates were similar in both settings only for antibodies to MSP1-19. Reduced exposure to infectious bites during the first six months in the high and seasonal transmission setting (Banfora) is a likely explanation of this similarity since only five malaria infections were detected during this period in Banfora, of which three occurred in the first rainy season in children aged above five months. The lack of acquisition of antibodies and the strong correlation of antibody levels between time points in Keur Soce indicates lack of exposure throughout the monitoring period. However in Banfora antibody levels were more variable from time point to time point, indicating that exposure to malaria was stimulating antibody production in some children, and furthermore that increases in antibody titres were often transient (Fig. S3).

Few studies have examined the role of anti-malaria antibodies in infants. We found that anti-AMA1-3D7 antibodies appeared as a marker of exposure (positively associated with the risk of febrile malaria) in our univariate analysis, which is consistent with the conclusions of Riley et al. [29]. Furthermore, adjusting for exposure using the exposure index attenuated the positive association between anti-AMA1 antibodies and increasing risk, consistent with anti-AMA1 antibodies acting as a marker of exposure in our study. Our results are also in keeping with the findings of previous studies in older children [30], [31]. Similarly, in our study, antibody titres to MSP1-19, MSP2-Dd2 and MSP3-3D7 were not associated with protection as in other infant cohort studies [16], [29], [32].

The apparent variation in results between infants and older children may be explained by the presence of a protective threshold. Murungi et al. and Rono et al. have analysed two independent cohorts of children and established protective threshold concentrations for some merozoite antigens [18], [19]. Even in a high transmission setting such as Burkina Faso, we found that antibody titres in the first six months of life for the four tested antigens were well below these protective threshold concentrations. Furthermore there was no association with protection. We conclude that these maternally-derived antibodies are not the protective mechanism. If the antibody levels are not protective in these infants then what is the basis of their apparent early and short-lived protection period against the clinical manifestation of malaria?

The possibilities are that other antibodies not measured in our work are related to protection [33], or that other antibody-mediated mechanisms not correlated with ELISA measurements are operating [34] or that other mechanisms operate such as nutritional factors [11], foetal haemoglobin [10], less infectious bites due to a reduced surface area [12], [35]. Previous data on passive transfer of antibodies from cord blood suggest that antibodies are involved [2].

## 6 Conclusion

Even at high intensity of malaria transmission, antibody levels to the tested *P. falciparum* merozoite antigens (AMA1-3D7, MSP1-19, MSP2-Dd2 and MSP3-3D7) remained low compared with the previously established protective threshold concentrations. In addition, the antibodies were not significantly associated with a reduced risk of malaria raising more questions on the basis of the early apparent protection against febrile malaria.

However, these results should be interpreted with caution because of the limitations of this study. The protective thresholds used in the present study have been established in cohorts including children more than 2 years old in addition to infants. In our cohort, the lack of any protective effect did not allow us to determine specific thresholds for this age group. Furthermore, the timing of the study, in which the first few months of life overlapped with the low malaria transmission season, was not ideal to assess the role of maternal antibodies at the higher titres seen in the first few months of life. However, if antibody thresholds are related to real biological phenomena than we would expect, the thresholds should be similar across study sites and age group. Hence the main contribution of our work is the demonstration that antibody levels in infants to selected merozoite antigens are well below previously defined protective thresholds, and therefore that these are unlikely to be responsible for mediating protection in vivo. Further work to identify protective antibody responses might include assessment of antibodies to other targets including red cell surface antigens, and functional assays in which the interaction of antibodies with immune cells is taken into account [34].

## Figures and Tables

**Table 1:** Multivariable models of risk of *P. falciparum* febrile malaria.

	Cox proportional hazards model			Negative binomial model		
Predictor	HR	95%CI	*p*	IRR	95%CI	*p*
Age	–	–	–	1.14	[1.09, 1.19]	<0.001
Anti-AMA1	1.14	[0.92, 1.42]	0.216	1.05	[0.92, 1.20]	0.504
Anti-MSP1	1.40	[1.09, 1.80]	0.008	1.14	[0.99, 1.31]	0.052
Anti-MSP2	0.89	[0.63, 1.27]	0.528	0.87	[0.72, 1.02]	0.079
Anti-MSP3	1.20	[0.63, 2.27]	0.578	0.93	[0.66, 1.33]	0.704
Foetal Hb rate (baseline)	0.97	[0.94, 0.99]	0.004	0.98	[0.97, 0.99]	0.041
Haemoglobin type						
AA	1	–	–	1	–	–
AS*	NA	–	0.121	NA	–	<0.001
AC	1.75	[0.88, 3.51]		1.44	[0.95,2.19]	
CC	0.28	[0.04, 2.19]		0.47	[0.15, 1.46]	
ITN use (pregnancy)						
Yes	1	–	–	1	–	–
No	1.05	[0.47, 2.36]	0.912	1.32	[0.78, 2.25]	0.305
Month of birth						
January	–	–	–	–	–	–
October	0.27	[0.07, 1.01]	0.269	0.49	[0.21, 1.16]	0.062
November	0.71	[0.34, 1.50]		0.91	[0.56, 1.50]	
December	0.70	[0.36, 1.39]		1.14	[0.74, 1.73]	
Season						
Dry season	1			1	–	–
Rains	9.39	[2.32, 37.99]	0.002	3.17	[2.17, 4.62]	<0.001
Malaria exposure index	1.10	[1.05, 1.15]	<0.001	1.08	[1.03, 1.12]	0.001

## Appendix A Supplementary data

Table S1 Correlation table of the different antigen-specific antibodies (Banfora cohort).

Table S2 Differences in seroprevalence of antibodies to merozoite antigens at baseline between Banfora (high transmission intensity) and Keur Soce (low transmission intensity).

Table S3 Average antibody decay rates in Banfora (High transmission intensity) and Keur Soce (Low transmission intensity). Excepting MSP1-19, average antibody decay rates were different in both the sites.

Table S4 Univariate models of risk of *P. falciparum* febrile malaria.
