# Supplementary material for: Dynamics and role of antibodies to Plasmodium falciparum merozoite antigens in children living in two settings with differing malaria transmission intensity
Source: Vaccine. 2016 Jan 2;34(1):160–6. doi: 10.1016/j.vaccine.2015.10.058 (PMC4683095; doi:10.1016/j.vaccine.2015.10.058)
Supplement: Table S1 — Correlation table of the different antigen-specific antibodies (Banfora cohort). [file mmc4.doc]

|  | AMA1-3D7 | MSP1-19 | MSP2-Dd2 | MSP3-3D7 |
| --- | --- | --- | --- | --- |
| AMA1-3D7 | 1 |  |  |  |
| MSP1-19 | 0.364 (p<0.001) | 1 |  |  |
| MSP2-Dd2 | 0.466 (p<0.001) | 0.472 (p<0.001) | 1 |  |
| MSP3-3D7 | 0.392 (p<0.001) | 0.214 (p<0.001) | 0.413 (p<0.001) | 1 |
