# Supplementary material for: Dynamics and role of antibodies to Plasmodium falciparum merozoite antigens in children living in two settings with differing malaria transmission intensity
Source: Vaccine. 2016 Jan 2;34(1):160–6. doi: 10.1016/j.vaccine.2015.10.058 (PMC4683095; doi:10.1016/j.vaccine.2015.10.058)
Supplement: Table S2 — Differences in seroprevalence of antibodies to merozoite antigens at baseline between Banfora (high transmission intensity) and Keur Soce (low transmission intensity). [file mmc5.docx]

|  | AMA1-3D7 | MSP1-19 | MSP2-Dd2 | MSP3-3D7 |
| --- | --- | --- | --- | --- |
| Banfora | 0.97 (0.87, 0.99) | 0.33 (0.19, 0.50) | 0.95 (0.83, 0.99) | 0.62 (0.45, 0.77) |
| Keur Soce | 0.80 (0.64, 0.91) | 0.25 (0.13, 0.41) | 0.58 (0.41, 0.73) | 0.23 (0.11, 0.38) |
|  | p=0.029* | p=0.465* | p<0.001* | P=0.001* |

*Two-sided Fisher’s Exact test
