# Supplementary material for: Dynamics and role of antibodies to Plasmodium falciparum merozoite antigens in children living in two settings with differing malaria transmission intensity
Source: Vaccine. 2016 Jan 2;34(1):160–6. doi: 10.1016/j.vaccine.2015.10.058 (PMC4683095; doi:10.1016/j.vaccine.2015.10.058)
Supplement: Table S3 — Average antibody decay rates in Banfora (High transmission intensity) and Keur Soce (Low transmission intensity). Excepting MSP1-19, average antibody decay rates were different in both the sites. [file mmc6.doc]

|  | Banfora cohort (site 1) | | Keur Soce cohort (site 2) | | Interaction age vs site |
| --- | --- | --- | --- | --- | --- |
| Antigen | Decay rate (log10 AU/month), 95 % CI | p value | Decay rate (log10 AU/month), 95 % CI | p value |
| AMA1-3D7 | -0.49 (-0.52, -0.45) | p<0.001 | -0.56 (-0.59, -0.53) | p<0.001 | p=0.003 |
| MSP1-19 | -0.32 (-0.37, -0.27) | p<0.001 | -0.27 (-0.32, -0.22) | p<0.001 | p=0.221 |
| MSP2-Dd2 | -0.47 (-0.50, -0.44) | p<0.001 | -0.23 (-0.27, -0.19) | p<0.001 | p<0.001 |
| MSP3-3D7 | -0.23 (-0.26, -0.20 ) | p<0.001 | -0.12 (-0.15, -0.10) | p<0.001 | p<0.001 |
