# Supplementary material for: Dynamics and role of antibodies to Plasmodium falciparum merozoite antigens in children living in two settings with differing malaria transmission intensity
Source: Vaccine. 2016 Jan 2;34(1):160–6. doi: 10.1016/j.vaccine.2015.10.058 (PMC4683095; doi:10.1016/j.vaccine.2015.10.058)
Supplement: Table S4 — Univariate models of risk of P. falciparum febrile malaria. [file mmc7.doc]

|  | Cox Proportional Hazards models | | | Negative binomial models | | |
| --- | --- | --- | --- | --- | --- | --- |
| Predictor | HR | 95% CI | P | IRR | 95% CI | P |
| Age | - | - | - | 1.14 | [1.12, 1.17] | <0.001 |
| Sex |  |  |  |  |  |  |
| Male | 1 | - | - | 1 | - | - |
| Female | 0.84 | [0.52, 1.35] | 0.472 | 0.76 | [0.50, 1.16] | 0.205 |
| Weight (baseline) | 0.95 | [0.64, 1.42] | 0.817 | 1.07 | [0.75, 1.51] | 0.722 |
| Length (baseline) | 1.02 | [0.92, 1.12] | 0.715 | 1.04 | [0.97, 1.12] | 0.300 |
| Fetal Hb rate (baseline) | 0.98 | [0.96, 1.001] | 0.072 | 0.99 | [0.97, 1.002] | 0.084 |
| Hemoglobin type |  |  |  |  |  |  |
| AA | 1 | - | - | 1 | - | - |
| AS* | NA | - | 0.198 | NA | - | <0.001 |
| AC | 1.81 | [0.97, 3.38] |  | 1.32 | [0.81,2.16] |  |
| CC | 0.36 | [0.05, 2.59] |  | 0.54 | [0.09, 3.11] |  |
| Anti-AMA1 | 1.34 | [1.11, 1.62] | 0.002 | 0.89 | [0.80, 0.98] | 0.015 |
| Anti-MSP1 | 1.44 | [1.19, 1.74] | <0.001 | 1.28 | [1.16, 1.42] | <0.001 |
| Anti-MSP2 | 1.27 | [0.95, 1.70] | 0.103 | 1.01 | [0.85, 1.21] | 0.911 |
| Anti-MSP3 | 1.28 | [0.70, 2.34] | 0.428 | 0.73 | [0.42, 1.26] | 0.260 |
| Seropositivity status to AMA1 |  |  |  |  |  |  |
| Seronegative | 1 | - | - | 1 | - | - |
| Seropositive | 1.62 | [0.92, 2.87] | 0.096 | 0.70 | [0.47, 1.05] | 0.087 |
| Seropositivity status to MSP1 |  |  |  |  |  |  |
| Seronegative | 1 | - | - | 1 | - | - |
| Seropositive | 2.99 | [1.55, 5.76] | 0.001 | 2.10 | [1.46, 3.03] | <0.001 |
| Seropositivity status to MSP2 |  |  |  |  |  |  |
| Seronegative | 1 | - | - | 1 | - | - |
| Seropositive | 2.003 | [1.09, 3.68] | 0.025 | 1.04 | [0.72, 1.50] | 0.842 |
| Seropositivity status to MSP3 |  |  |  |  |  |  |
| Seronegative | 1 | - | - | 1 | - | - |
| Seropositive | 1.39 | [0.76, 2.56] | 0.290 | 0.65 | [0.41, 1.03] | 0.064 |
| Month of birth |  |  |  |  |  |  |
| January | 1 | - | - | 1 | - | - |
| October | 0.19 | [0.06, 0.64] | 0.012 | 0.21 | [0.07, 0.64] | 0.002 |
| November | 0.44 | [0.23, 0.83] |  | 0.45 | [0.27, 0.73] |  |
| December | 0.53 | [0.29, 0.96] |  | 0.66 | [0.41, 1.06] |  |
| EPI status (baseline) |  |  |  |  |  |  |
| Up to date | 1 | - | - | 1 | - | - |
| Not up to date | 0.94 | [0.45, 1.96] | 0.862 | 1.06 | [0.60, 1.86] | 0.845 |
| Age mother (baseline) | 1.04 | [1.001, 1.08] | 0.042 | 1.02 | [0.99, 1.06] | 0.135 |
| Gravidity status |  |  |  |  |  |  |
| Primigravidae | 1 | - | - | 1 | - | - |
| Multigravidae | 1.12 | [0.61, 2.06] | 0.709 | 1.08 | [0.61, 1.89] | 0.797 |
| ITN use (pregnancy) |  |  |  |  |  |  |
| Yes | 1 | - | - | 1 | - | - |
| No | 1.24 | [0.61, 2.50] | 0.550 | 1.35 | [0.75, 2.45] | 0.320 |
| IPTp courses |  |  |  |  |  |  |
| 0 | 1 | - | - | 1 | - | - |
| 1 | 1.47 | [0.49, 4.41] | 0.639 | 1.70 | [0.71, 4.09] | 0.149 |
| 2 or 3 | 1.15 | [0.41, 3.19] |  | 1.07 | [0.47, 2.42] |  |
| Education level (mother) |  |  |  |  |  |  |
| None | 1 | - | - | 1 | - | - |
| Primary | 1.52 | [0.92, 2.51] | 0.031 | 1.38 | [0.91, 2.09] | <0.001 |
| Secondary or above | 0.45 | [0.18, 1.15] |  | 0.31 | [0.15, 0.64] |  |
| Zone of residence |  |  |  |  |  |  |
| Rural | 1 | - | - | 1 | - | - |
| Urban | 0.31 | [0.16, 0.61] | <0.001 | 0.30 | [0.16, 0.56] | <0.001 |
| Mixed | 1.32 | [0.78, 2.23] |  | 1.22 | [0.80, 1.84] |  |
| Season |  |  |  |  |  |  |
| Dry season | 1 |  |  | 1 | - | - |
| Rains | 8.28 | [2.18, 31.44] | 0.002 | 4.32 | [2.88, 6.49] | <0.001 |
| Malaria Exposure index | 1.10 | [1.05, 1.14] | <0.001 | 1.08 | [1.03, 1.13] | 0.001 |
